# Performance of Large Language Models on Cognitive Aptitude Testing: A Multi-Run Evaluation on the German Medical School Admission Test (TMS)

**DOI:** 10.3390/ejihpe16020023

**Published:** 2026-02-12

**Authors:** Henrik Stelling, Armin Kraus, Gerrit Grieb, Ibrahim Güler

**Affiliations:** 1 Practices for Nuclear Medicine, Rubensstraße 125, 12157 Berlin, Germany; henrik.stelling@klinikumevb.de; 2 Department of Plastic, Aesthetic and Hand Surgery, Otto-von-Guericke University, 39120 Magdeburg, Germany; armin.kraus@med.ovgu.de; 3 Department of Plastic Surgery and Hand Surgery, Gemeinschaftskrankenhaus Havelhoehe, Kladower Damm 221, 14089 Berlin, Germany; gerritgrieb@gmx.de; 4 Department of Plastic Surgery and Hand Surgery, Burn Center, Medical Faculty, RWTH Aachen University, Pauwelsstrasse 30, 52074 Aachen, Germany; 5 Department of Health Management, Friedrich-Alexander-Universität Erlangen-Nürnberg (FAU), Lange Gasse 20, 90403 Nürnberg, Germany

**Keywords:** large language models, artificial intelligence, Test for Medical Studies (TMS), medical school admission test, medical education, cognitive aptitude, clinical reasoning, medical student selection, psychometrics, benchmarking

## Abstract

Background and Objectives: Large language models (LLMs) have demonstrated high performance on knowledge-based medical examinations but their capabilities on cognitive aptitude tests emphasizing reasoning and abstraction remain underexplored. The Test for Medical Studies (TMS), a German medical school admission test, provides a standardized framework to examine these capabilities. This study aimed to evaluate the performance and consistency of multiple LLMs on text-based and visual-analytic TMS items. Materials and Methods: Eight contemporary LLMs, comprising proprietary and open-source systems, were evaluated using a multi-run design on standardized TMS items spanning text-based and visual-analytic cognitive domains. Results: Mean accuracy remained substantially below levels typically reported for knowledge-based medical examinations, with marked performance differences between text-based and visual-analytic subtests. Open-source models performed competitively compared with proprietary systems. Inter-run reliability was heterogeneous, indicating notable variability across repeated evaluations. Conclusions: Current LLMs show limited and domain-dependent performance on cognitive aptitude tasks relevant to medical school admission. High accuracy on knowledge-based examinations does not translate into stable performance on aptitude tests emphasizing fluid intelligence. The observed modality-dependent performance patterns and inter-run variability highlight the importance of differentiated, multi-run evaluation strategies when assessing LLMs for applications in medical education.

## 1. Introduction

### 1.1. LLM Performance on Knowledge-Based Medical Examinations

Large language models (LLMs)*, along with emerging Multimodal LLMs (MLLMs) that extend these capabilities to modalities including visual inputs, have recently demonstrated striking performance on knowledge-based medical examinations, prompting intense discussion about their potential role in medical education and assessment. Multiple studies evaluating advanced LLMs on medical licensing examinations such as the United States Medical Licensing Examination (USMLE) have reported accuracy rates ranging from 81% to 95%, in some cases exceeding the average performance of medical students and approaching expert-level benchmarks (Kung et al., 2023; Mavrych et al., 2025; Gilson et al., 2023; Nori et al., 2023; Y. Chen et al., 2024; Bicknell et al., 2024). Comparable findings have been reported for the German medical licensing examination (Second State Examination, M2), where GPT-4 achieved accuracy rates of approximately 85% and ranked between the 92nd and 99th percentile of human test-takers (Meyer et al., 2024). These results have fueled expectations that LLMs may serve as powerful tools for learning support, formative assessment, and exam preparation. However, such examinations primarily assess crystallized intelligence, defined as the retrieval and application of accumulated domain knowledge (Zong et al., 2024; Elkin et al., 2025).

* For terminological consistency, the term LLMs is used throughout this manuscript to refer to all evaluated transformer-based models, including multimodal variants.

### 1.2. Crystallized Versus Fluid Intelligence in Medical Assessment

In contrast, medical school admission processes often rely on cognitive aptitude tests designed to measure fluid intelligence, namely the capacity to reason, abstract, and solve novel problems independent of prior training. In Germany, the Test for Medical Studies (Test für Medizinische Studiengänge, TMS) represents the central standardized instrument for this purpose. Developed by the Institute for Test and Talent Research (Institut für Test- und Begabungsforschung, ITB) in Bonn since 1978 (Trost, 1992), the TMS is currently used by approximately 36–37 German medical faculties, depending on the counting methodology, to inform admission decisions (TMS, n.d.; ITB-Academic Tests, n.d.). The examination does not require factual medical knowledge, instead all information necessary to solve the tasks is provided within the test items themselves. Its objective is to assess general learning potential rather than acquired knowledge, encompassing verbal, numerical, memory-related, and visual-spatial reasoning across multiple subtests (Kadmon & Kadmon, 2016; Zimmerhofer et al., 2019).

The relevance of the TMS for undergraduate medical education is well established. Meta-analytic evidence on subject-specific aptitude tests indicates particularly high predictive validity for human medicine, with reported coefficients around ρ ≈ 0.50, placing medicine among the disciplines with the strongest observed effects. In addition, TMS performance provides substantial incremental explanatory power beyond secondary school grades, increasing the explained variance in academic outcomes by approximately 8 percentage points (ΔR^2^ = 0.081) (Hell et al., 2007; Schult et al., 2019). Importantly, the cognitive constructs measured by the TMS differ fundamentally from those targeted by knowledge-based licensing examinations. Whereas licensing exams may allow artificial intelligence systems to leverage pattern matching against extensive training corpora, aptitude tests intentionally minimize reliance on memorized facts and instead require novel reasoning under strict constraints.

### 1.3. Research Gap and Educational Relevance

Understanding LLM performance on cognitive aptitude tests serves multiple educational purposes. First, if LLMs demonstrate high and stable performance, this could enable their use as tools for aptitude training, helping students develop reasoning strategies through interactive practice. Conversely, if models approach human-level performance, this raises concerns about examination integrity, as AI-assisted responses could undermine the validity of aptitude-based admission procedures, the present study addresses both perspectives by evaluating not only aggregate accuracy but also reasoning stability, which is critical for tutoring applications (where consistent feedback is essential) and security considerations (where variable performance may indicate detectability).

Despite extensive benchmarking of LLMs on knowledge-based medical examinations, their performance on such cognitive aptitude tests remains largely unexplored. This gap is particularly relevant because aptitude tests impose different and potentially more demanding cognitive requirements. Prior work on isolated reasoning and visual-spatial benchmarks suggests that LLM performance on aptitude-style tasks is substantially limited and unstable, with accuracy often failing to reliably exceed random baselines in certain domains (Li et al., 2025).

The present study addresses this gap by evaluating eight contemporary LLMs on standardized TMS items. Specifically, this study aimed to:

(1) evaluate model accuracy across verbal-conceptual and visual-spatial domains using a multi-run design
(2) assess inter-run reliability and reasoning stability, which are critical for educational and assessment-related applications
(3) identify performance differences between proprietary and open-source models
(4) compare aptitude-based performance patterns with previously reported results on knowledge-based medical examinations

## 2. Materials and Methods

### 2.1. Study Design

We conducted a multi-run evaluation of eight contemporary LLMs using standardized items from the TMS, Germany’s national cognitive aptitude test for medical school admission. Each model completed the assigned item set across five independent runs to assess both accuracy and reasoning stability. In total, 5400 observations were generated using a split-modality design: seven models were evaluated on the full item set (144 items × 5 runs = 5040 observations), while one model (DeepSeek V3) was evaluated on a reduced text-only subset (72 items × 5 runs = 360 observations) due to technical limitations in visual input processing.

### 2.2. AI Models

Eight state-of-the-art LLMs were evaluated, comprising five proprietary and three open-source models.

- Proprietary models (*n* = 5): GPT-5 (OpenAI, San Francisco, CA, USA), Grok 4 (xAI, San Francisco, CA, USA), Claude Sonnet 4.5 (Anthropic, San Francisco, CA, USA), Gemini 2.5 (Google LLC, Mountain View, CA, USA), and ERNIE 4.5 Turbo (Baidu, Beijing, China).
- Open-source models (*n* = 3): DeepSeek V3 (DeepSeek, Hangzhou, China), Qwen3-Max (Alibaba Cloud, Hangzhou, China), and Mistral Medium 3.1 (Mistral AI, Paris, France).

Note: All models were accessed exclusively via their official web interfaces during October 2025 using default settings, without any additional prompt engineering or parameter modifications.

Mistral Medium 3.1 is formally a proprietary model. However, it is categorized here alongside open-source models to reflect Mistral’s established tradition of open-source releases and to maintain conceptual balance between the model groups analyzed.

### 2.3. Examination Material and TMS Subtests

The complete TMS comprises 184 scored items distributed across multiple subtests covering verbal, numerical, spatial-figural, and memory-related cognitive domains. For the present study, six subtests focusing on reasoning, abstraction, and analytical processing were selected. Subtests primarily targeting short-term memory (Learning Figures, Learning Facts) or sustained attention and processing speed (Concentrated Work) were excluded, as these dimensions are less suitable for evaluating LLM reasoning behavior.

The examination items used in this study were drawn from previously administered original TMS questions with officially published solutions. These materials originate from historical TMS examinations and are available through a combination of publicly accessible online resources and officially distributed print preparation materials. All questions were used in their original form without modification of wording, answer options, or scoring keys.

An overview of the included subtests, their cognitive focus, and input modality is provided in Table 1. Detailed subtest descriptions and officially released example items are publicly available through ITB preparation materials and institutional resources (TMS, n.d.; ITB-Academic Tests, n.d.; Zimmerhofer et al., 2019).

### 2.4. Modality-Based Evaluation Strategy

Models were evaluated according to their supported input modalities.

- Standard item set (144 items): Administered to seven models (GPT-5, Grok 4, Claude Sonnet 4.5, Gemini 2.5, ERNIE 4.5 Turbo, Qwen3-Max, and Mistral Medium 3.1). These models processed all six subtests listed in Table 1, including tasks requiring visual or visual-analytic reasoning.
- Text-only item set (72 items): Administered to DeepSeek V3. At the time of evaluation, image input in the evaluated model version was limited to optical character recognition (OCR) and did not support visual or visual-analytic reasoning. Consequently, the three subtests requiring such capabilities (Muster zuordnen, Schlauchfiguren, Diagramme und Tabellen) were excluded, and DeepSeek V3 was evaluated exclusively on the remaining text-based subtests.
- Aggregate text-based accuracy values reflect performance across all eight models, whereas visual-analytic accuracy values are based on the seven multimodal models.

### 2.5. Prompting Procedure and Data Collection

A standardized zero-shot prompting strategy was applied uniformly across all models and runs. Each prompt presented the task context, the full question stem, and five answer options (A–E), explicitly instructing the model to return only the single-letter answer corresponding to the selected option, without any explanation or justification (López Espejel et al., 2023).

Each model completed five independent runs, with each run initiated in a new browser session to minimize potential carryover or context effects. No additional instructions, examples, or reasoning cues were provided. Model responses were recorded as categorical outputs (A–E) and scored according to standard TMS conventions, awarding +1 point for correct answers and 0 points for incorrect answers, without penalties for guessing.

### 2.6. Statistical Analysis

All statistical analyses were performed using Python 3.12 with standard statistical and data visualization libraries.

Descriptive performance metrics included mean accuracy across runs and corresponding 95% confidence intervals.

Inter-run reliability and reasoning stability were assessed using Cohen’s kappa (κ), calculated for all ten possible run pairs per model. Interpretation followed established benchmarks: κ < 0.21 (slight), 0.21–0.40 (fair), 0.41–0.60 (moderate), 0.61–0.80 (substantial), and >0.80 (almost perfect) (Cohen, 1960; Landis & Koch, 1977).

Pairwise performance differences between models were evaluated using McNemar’s test for paired nominal data. To control for multiple comparisons across 28 model pairs, a Bonferroni-corrected significance threshold of α = 0.0018 was applied. Comparisons involving DeepSeek V3 were restricted to the shared 72-item text-based subset.

## 3. Results

### 3.1. Overall Performance

All eight LLMs completed the evaluation across five independent runs, yielding a total of 5400 observations. Seven models were evaluated on the full 144-item dataset comprising text-based and visual-analytic TMS subtests, whereas DeepSeek V3 was evaluated on a reduced 72-item text-only subset due to modality constraints. All models achieved accuracies above the theoretical chance level for five-option multiple-choice items (20%).

### 3.2. Model-Level Performance and Ranking

**Figure 1:**
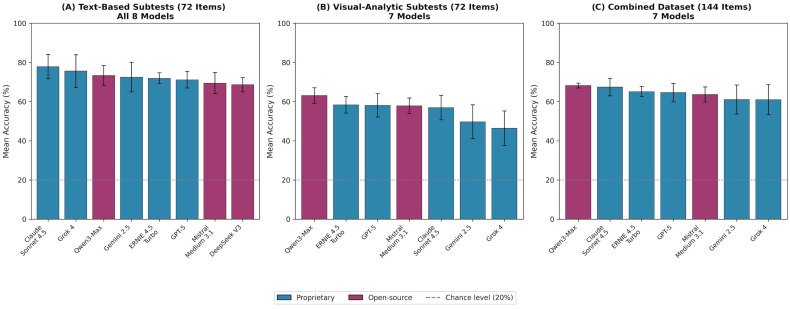
Model Performance across Different Modalities on Standardized TMS Items.

To enable equitable comparison across evaluation conditions, model performance was analyzed separately for text-based items (72 items, all eight models), visual-analytic items (72 items, seven models excluding DeepSeek V3), and the combined dataset (144 items, seven models). Results are visualized in Figure 1 and summarized in Table 2.

#### 3.2.1. Text-Based Subtests (72 Items)

On the shared text-based subset, Claude Sonnet 4.5 achieved the highest mean accuracy, followed by Grok 4 and Qwen3-Max. DeepSeek V3 achieved the lowest accuracy among all eight models.

#### 3.2.2. Visual-Analytic Subtests (72 Items)

On visual-analytic items, Qwen3-Max achieved the highest accuracy, followed by ERNIE 4.5 Turbo. The lowest accuracies were observed for Grok 4 and Gemini 2.5. Across all evaluated models, mean accuracy on text-based items was 72.5%, whereas mean accuracy on visual-analytic items was 55.8% among the seven multimodal models, corresponding to a difference of 16.7 percentage points.

#### 3.2.3. Combined Dataset (144 Items)

On the full 144-item dataset, Qwen3-Max achieved the highest mean accuracy, followed by Claude Sonnet 4.5 and ERNIE 4.5 Turbo. The lowest mean accuracies were observed for Grok 4 and Gemini 2.5. Across all seven multimodal models, the aggregate mean accuracy on the combined 144-item dataset was 64.4%.

#### 3.2.4. Inter-Run Variability

Inter-run variability differed across models and modalities. On text-based items, ERNIE 4.5 Turbo showed the lowest variability, whereas Grok 4 exhibited the highest. On visual-analytic items, variability was generally higher, with Grok 4 and Gemini 2.5 showing particularly unstable performance. This variability reflects fluctuations in aggregate accuracy across runs and does not capture item-level response consistency, which is assessed separately using Cohen’s κ.

### 3.3. Proprietary Versus Open-Source Models

When grouped by licensing paradigm using the combined 144-item dataset (Panel C), open-source models (*n* = 2) achieved a mean accuracy of 65.9% (range 63.6–68.2%), while proprietary models (*n* = 5) achieved a mean accuracy of 63.8% (range 61.0–67.4%).

### 3.4. Inter-Run Reliability and Reasoning Stability

Inter-run reliability, assessed using Cohen’s κ across all ten run pairs per model, is summarized in Table 3. Mean κ values ranged from 0.208 to 0.676. DeepSeek V3 showed the highest mean κ (0.676), followed by Mistral Medium 3.1 (0.452) and Qwen3-Max (0.424). The remaining models exhibited lower κ values, spanning fair to slight agreement according to established benchmarks.

### 3.5. Pairwise Model Comparisons

Pairwise performance differences were evaluated using McNemar’s test with Bonferroni correction for multiple comparisons (adjusted α = 0.0018). Of the 28 possible pairwise comparisons, one comparison reached statistical significance after correction: Claude Sonnet 4.5 versus DeepSeek V3 (χ^2^ = 10.29, *p* = 0.0013; odds ratio = 13.0). This comparison was restricted to the shared 72-item text-only subset. Six additional comparisons reached nominal significance (*p* < 0.05), including one comparison with *p* < 0.01, but none survived correction (Table 4).

**Figure 2:**
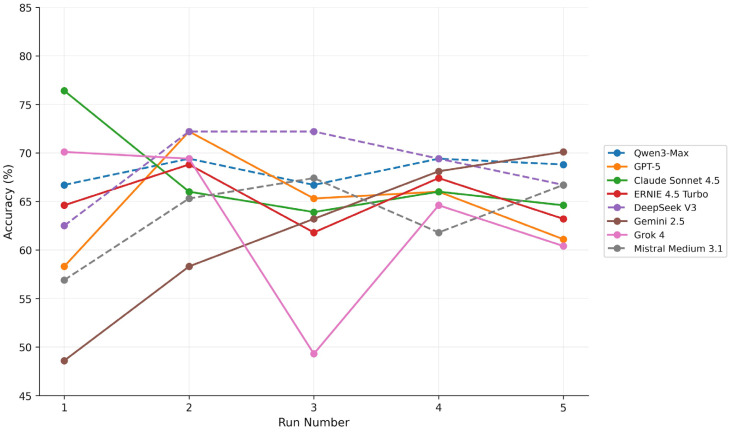
Run-specific accuracy across five independent evaluations.

Figure 2 illustrates run-specific accuracy values across five independent runs for each model, providing a descriptive visualization of inter-run variability underlying the summary statistics reported above.

Run-specific accuracy (%) across five independent runs for each model is represented in Figure 2. Each point represents the aggregate accuracy of a single run. The figure illustrates inter-run variability underlying both the coefficient of variation (CV) and the reported range in Table 2.

## 4. Discussion

### 4.1. Main Findings

This study provides a systematic evaluation of LLM performance on the TMS, a standardized cognitive aptitude test used for medical school admission in Germany. In contrast to the near-expert performance previously reported for knowledge-based medical licensing examinations, all evaluated models achieved only moderate accuracy on TMS items (Kung et al., 2023; Mavrych et al., 2025; Gilson et al., 2023; Nori et al., 2023; Y. Chen et al., 2024; Bicknell et al., 2024; Meyer et al., 2024). Mean performance ranged from 61.0% to 68.2%, with an aggregate mean of 64.4% (Table 2 Panel C, Figure 1), indicating a substantially lower performance ceiling on aptitude tasks emphasizing reasoning, abstraction, and problem solving.

A key finding of the modality-stratified analysis is the substantial performance difference between text-based and visual-analytic items. Across all evaluated models, accuracy on text-based subtests was higher (mean 72.5%) than on visual-analytic subtests, which were completed by the seven multimodal models (mean 55.8%), corresponding to an average difference of 16.7 percentage points. Importantly, this gap does not necessarily indicate that visual-analytic items are inherently more difficult in an absolute sense, rather, it suggests that current LLM architectures are better aligned with linguistic processing demands than with visual-spatial cognitive requirements. This interpretation is consistent with the differential training emphasis of transformer-based models, which are predominantly optimized on text corpora.

Beyond aggregate accuracy, a central finding of this study is the limited reasoning stability of current LLMs. While overall performance differences between models were relatively small, inter-run reliability (Table 3) varied considerably, with most systems demonstrating only fair to moderate agreement across repeated evaluations. These findings highlight that single-run benchmark results may not adequately capture model behavior in aptitude-focused settings.

Consistent with this observation, pairwise model comparisons revealed only limited statistically significant differences after correction for multiple testing (Table 4), reflecting both the compressed performance range across models and the conservative nature of the applied Bonferroni adjustment under conditions of modest statistical power.

### 4.2. Crystallized Versus Fluid Intelligence

The discrepancy between strong performance on medical licensing examinations (Liu et al., 2024) and weaker performance on the TMS reflects a fundamental distinction between crystallized and fluid intelligence. Knowledge-based examinations predominantly assess crystallized intelligence, relying on the retrieval and application of previously acquired factual knowledge. LLMs appear well suited to this domain, likely benefiting from extensive exposure to medical and scientific texts during training.

In contrast, the TMS is explicitly designed to assess fluid intelligence, requiring examinees to solve novel problems under constrained conditions without reliance on prior medical knowledge. The moderate accuracy levels observed across all models suggest that current LLM architectures struggle to generalize reasoning strategies to such tasks, particularly when memorization or pattern matching provides limited advantage. This observation is consistent with recent findings from abstract and visual-spatial reasoning benchmarks, which similarly report unstable or inconsistent model performance (Yang et al., 2025; Ilić & Gignac, 2024).

### 4.3. Visual-Analytic Items vs. Text-Based Items

The modality-stratified analysis further illuminates these architectural limitations. Visual-analytic subtests such as Schlauchfiguren (mental rotation of three-dimensional tube figures) and Muster zuordnen (pattern matching requiring precise perceptual discrimination) present particular challenges for current multimodal architectures. Unlike text-based reasoning tasks where models can leverage linguistic pattern recognition, these subtests require genuine spatial manipulation and fine-grained visual comparison. The substantially lower accuracy observed on visual-analytic items (mean 55.8% vs. 72.5% on text-based items) suggests that current vision encoders struggle to maintain coherent three-dimensional mental representations during rotation operations and to extract diagnostic features from complex graphical stimuli with sufficient precision. Importantly, this performance gap reflects differential alignment between LLM architectures and distinct cognitive demands rather than a simple difficulty gradient. These findings indicate that improvements in visual-spatial reasoning may require architectural advances specifically targeting spatial representation and manipulation capabilities.

### 4.4. Open-Source Versus Proprietary Models

An important finding of this study is the competitive performance of open-source models. Qwen3-Max ranked among the highest-performing systems on the combined dataset. These results challenge the assumption that proprietary architectures inherently provide superior reasoning capabilities on complex cognitive tasks. Evidence from clinical reasoning benchmarks further supports this observation, demonstrating that frontier open-source LLMs can match, and in selected metrics slightly exceed, the performance of proprietary models on complex diagnostic tasks (Buckley et al., 2025).

From the perspective of medical education research, the strong performance of open-source models is noteworthy, as such systems offer advantages in transparency, reproducibility, and data governance. Nevertheless, competitive accuracy alone does not compensate for the observed limitations in reasoning stability, which were present across both proprietary and open-source model classes.

### 4.5. Reliability and Implications for Medical Education

Inter-run reliability represents a critical consideration for educational applications. While some models exhibited relatively stable aggregate scores across repeated runs, item-level agreement remained limited for most systems (Table 2 and Table 3; Figure 2). This indicates that identical questions may elicit different responses upon re-evaluation, even under standardized prompting conditions. Single-run evaluations may substantially overestimate or underestimate model capabilities (Alvarado Gonzalez et al., 2025).

For applications related to medical school admission preparation or aptitude training, such variability constrains practical usability. If an AI system is used as a tutor, inconsistent reasoning across attempts may undermine the learning process, as students cannot reliably rely on the model to reinforce correct logic patterns. The present findings therefore suggest that, despite promising accuracy levels, improvements in reasoning stability are required before LLMs can be reliably integrated into aptitude-focused educational settings.

### 4.6. Limitations

Several limitations should be considered when interpreting these results.

1. Restricted evaluation scope for DeepSeek V3: DeepSeek V3 was evaluated on a reduced, text-only subset of TMS items due to modality constraints, limiting direct comparability with multimodal models on visual and visual-analytic subtests.
2. Zero-shot prompting paradigm: A standardized zero-shot prompting strategy was employed to reflect realistic user interaction. While this enhances ecological validity, alternative prompting approaches (e.g., structured reasoning prompts) may yield different performance profiles and were not examined in this study.
3. Use of historical examination material: The TMS items used in this evaluation were drawn from previously administered examinations with officially published solutions. As these materials are available through public and print-based preparation resources, it cannot be excluded that some items were included in the training corpora of the evaluated models. However, this limitation applies broadly across models and does not explain the observed differences in inter-run reliability.
4. Limited control over generation parameters: All models were accessed via official web interfaces without direct control over temperature or other decoding parameters. As a result, internal sampling strategies may have varied across models and over time, potentially contributing to inter-run variability.
5. Exclusion of specific cognitive subtests: Subtests primarily targeting short-term memory (Learning Figures, Learning Facts) and sustained attention/processing speed (Concentrated Work) were excluded. Consequently, the present analysis does not represent a full benchmark of TMS performance across all cognitive domains. However, these subtests are conceptually and technically ill-suited for evaluation with current LLM architectures.
6. Absence of direct human performance comparison: No direct comparison with human TMS performance was conducted, precluding assessment of whether LLM performance approaches, matches, or exceeds typical applicant outcomes. While human test participants receive both absolute scores and percentile ranks, admission decisions are primarily based on the percentile ranks (Test für Medizinische Studiengänge [TMS], n.d.). Since the present study evaluated a selected subset of items without access to a representative human reference cohort, a valid mapping of LLM performance to human standard values or percentile ranks was not feasible. Accordingly, the results are intended to characterize relative performance patterns across models rather than direct equivalence to human test-takers.
7. Dynamic nature of deployed LLM systems: The evaluated models are continuously updated by their providers, and their underlying parameters, training data, and inference behavior may change over time (L. Chen et al., 2023). As a result, performance estimates may vary across evaluation dates. While offline evaluation of fixed model checkpoints would improve reproducibility, such an approach would not reflect the real-world usage scenario of most end users, who typically interact with continuously updated web-based systems and lack the computational resources or technical expertise required for local deployment.
8. Choice of reliability metric: Cohen’s κ was selected as the primary inter-run reliability measure because it quantifies categorical agreement between paired runs while correcting for chance agreement, which is appropriate for evaluating whether models produce consistent responses to identical items. Importantly, κ captures response consistency rather than reasoning quality; high agreement may reflect stable but incorrect reasoning patterns, while low agreement indicates response variability regardless of underlying accuracy. Other studies may prefer Krippendorff’s α to estimate overall reliability across multiple runs.

## 5. Conclusions

Current LLMs show limited and domain-dependent performance on cognitive aptitude tasks relevant to medical school admission and education. High accuracy on knowledge-based medical examinations, primarily reflecting crystallized intelligence, does not directly translate into stable performance on aptitude tests emphasizing fluid intelligence, such as reasoning and abstraction.

The compressed performance range across models and the limited number of significant pairwise differences suggest shared constraints in current architectures when reasoning beyond memorized information.

The observed inter-run variability highlights the importance of multi-run evaluation strategies when assessing LLMs for applications in medical education and educational assessment.

From a theoretical perspective, these findings advance understanding of the boundaries between crystallized and fluid intelligence in artificial systems, demonstrating that strong performance on knowledge retrieval tasks does not generalize to novel reasoning demands requiring spatial manipulation or abstract problem-solving. The observed modality-dependent performance patterns further suggest that current architectures are differentially suited to linguistic versus visual-spatial cognitive demands, rather than exhibiting uniform reasoning capabilities across domains. Practically, the results inform governance considerations for AI deployment in medical education: current LLMs should not be relied upon as standalone tools for aptitude training or admission preparation without substantial human oversight, and the observed instability raises questions about both tutoring efficacy and examination security in high-stakes assessment contexts.

Future studies should evaluate multimodal performance on visual-spatial subtests and consider synchronized human–AI benchmarking under comparable testing conditions.

## Figures and Tables

**Table 1:** Overview of TMS subtests included in the evaluation, categorized by cognitive domain and input modality.

Subtest (German Original)	Cognitive Domain & Task Description	N Items	Modality
Medizinisch- naturwissenschaftliches Grundverständnis	Deductive reasoning based on short medical–scientific texts without requiring prior knowledge	24	Text
Quantitative und formale Probleme	Mathematical and logical reasoning embedded in biomedical contexts	24	Text
Textverständnis	Synthesis and interpretation of complex scientific texts	24	Text
Diagramme und Tabellen	Visual-analytic reasoning involving graphs, curves, and tabulated data	24	Visual
Muster zuordnen	Perceptual precision and error detection in complex graphical representations	24	Visual
Schlauchfiguren	Spatial reasoning requiring mental rotation of three-dimensional objects	24	Visual
Total included items		144	

**Table 2:** Model Performance on Standardized TMS Items by Modality.

**Panel A: Text-Based Subtests (72 Items)—All 8 Models**								
**Rank**	**Model**	**Type**	**N**	**Mean** **Accuracy (%)**	**95% CI**	**SD**	**CV (%)**	**Range**
1	Claude Sonnet 4.5	Prop.	72	77.8	71.6–84.0	7.08	9.1	18
2	Grok 4	Prop.	72	75.6	67.2–83.9	9.55	12.6	26
3	Qwen3-Max	Open	72	73.3	68.3–78.4	5.76	7.9	15
4	Gemini 2.5	Prop.	72	72.5	65.0–80.0	8.53	11.8	16
5	ERNIE 4.5 Turbo	Prop.	72	71.9	69.2–74.7	3.17	4.4	8
6	GPT-5	Prop.	72	71.1	66.9–75.4	4.85	6.8	12
7	Mistral Medium 3.1	Open	72	69.4	64.1–74.8	6.13	8.8	15
8	DeepSeek V3	Open	72	68.6	65.0–72.2	4.12	6.0	9
**Panel B: Visual-Analytic Subtests (72 Items)—7 Models**								
**Rank**	**Model**	**Type**	**N**	**Mean** **Accuracy (%)**	**95% CI**	**SD**	**CV (%)**	**Range**
1	Qwen3-Max	Open	72	63.1	59.1–67.0	4.46	7.1	11
2	ERNIE 4.5 Turbo	Prop.	72	58.3	54.1–62.6	4.81	8.2	11
3	GPT-5	Prop.	72	58.1	52.1–64.0	6.76	11.6	15
4	Mistral Medium 3.1	Open	72	57.8	53.8–61.8	4.56	7.9	12
5	Claude Sonnet 4.5	Prop.	72	56.9	50.7–63.2	7.15	12.6	18
6	Gemini 2.5	Prop.	72	49.7	41.1–58.3	9.79	19.7	25
7	Grok 4	Prop.	72	46.4	37.6–55.2	10	21.5	22
**Panel C: Combined Dataset (144 Items)—7 Models**								
**Rank**	**Model**	**Type**	**N**	**Mean** **Accuracy (%)**	**95% CI**	**SD**	**CV (%)**	**Range**
1	Qwen3-Max	Open	144	68.2	66.9–69.4	1.42	2.1	2
2	Claude Sonnet 4.5	Prop.	144	67.4	62.9–71.9	5.13	7.6	12
3	ERNIE 4.5 Turbo	Prop.	144	65.1	62.6–67.7	2.88	4.4	6
4	GPT-5	Prop.	144	64.6	59.9–69.2	5.29	8.2	13
5	Mistral Medium 3.1	Open	144	63.6	59.8–67.4	4.3	6.8	10
6	Gemini 2.5	Prop.	144	61.1	53.7–68.5	8.43	13.8	20
7	Grok 4	Prop.	144	61.0	53.3–68.6	8.75	14.3	19

Note: DeepSeek V3 was evaluated exclusively on text-based subtests due to modality constraints and is therefore included only in Panel A. Abbreviations: N—number of items; 95% CI—95% confidence interval; SD—standard deviation; CV—coefficient of variation; Prop.—proprietary; Range—difference between highest and lowest run accuracy in percentage points.

**Table 3:** Inter-Run Reliability by Model.

Model	Mean κ	Min κ	Max κ	Interpretation
DeepSeek V3	0.676	0.543	0.788	Substantial
Mistral Medium 3.1	0.452	0.363	0.567	Moderate
Qwen3-Max	0.424	0.234	0.617	Moderate
Gemini 2.5	0.355	0.143	0.724	Fair
Claude Sonnet 4.5	0.347	0.223	0.567	Fair
Grok 4	0.340	0.060	0.671	Fair
GPT-5	0.307	0.040	0.487	Fair
ERNIE 4.5 Turbo	0.208	0.025	0.338	Slight

κ—Cohen’s kappa. Interpretation per Landis & Koch (Cohen, 1960; Landis & Koch, 1977).

**Table 4:** Pairwise Comparisons (McNemar’s Test).

Model A	Model B	N	χ^2^	*p*-Value	OR	Sig
Claude Sonnet 4.5	DeepSeek V3 ^†^	72	10.29	0.0013	13.0	***
Claude Sonnet 4.5	Gemini 2.5	144	8.00	0.0047	3.0	**
ERNIE 4.5 Turbo	Gemini 2.5	144	6.43	0.0112	2.5	*
Qwen3-Max	Gemini 2.5	144	5.49	0.0191	2.15	*
DeepSeek V3 ^†^	Grok 4	72	5.33	0.0209	0.2	*
GPT-5	Gemini 2.5	144	4.83	0.0280	2.18	*
ERNIE 4.5 Turbo	DeepSeek V3 ^†^	72	4.00	0.0455	3.0	*

Only pairwise comparisons with *p* < 0.05 are shown; 21 additional comparisons were non-significant. Abbreviations: N—number of shared items; χ^2^—McNemar chi-square statistic; OR—odds ratio; Sig—significance level. *** *p* < 0.0018 (Bonferroni-corrected); ** *p* < 0.01; * *p* < 0.05. ^†^ Comparisons involving DeepSeek V3 utilized only the 72 shared text-based items.

## Data Availability

The raw data presented in this study are available on request from the corresponding author.

## Abbreviations

The following abbreviations are used in this manuscript:

AI	Artificial Intelligence
CI	Confidence Interval
CV	Coefficient of Variation
ITB	Institute for Test and Talent Research (Institut für Test- und Begabungsforschung)
κ	Cohen’s Kappa
LLM	Large Language Model
M2	Second State Examination (German medical licensing examination)
MLLM	Multimodal Large Language Model
OCR	Optical Character Recognition
SD	Standard Deviation
TMS	Test for Medical Studies (Test für Medizinische Studiengänge; “Medizinertest”)
USMLE	United States Medical Licensing Examination

## References

[B1-ejihpe-16-00023] Alvarado Gonzalez M. A., Bruno Hernandez M., Peñaloza Perez M. A., Lopez Orozco B., Cruz Soto J. T., Malagon S. (2025). Do repetitions matter? Strengthening reliability in LLM evaluations. arXiv.

[B2-ejihpe-16-00023] Bicknell B. T., Butler D., Whalen S., Ricks J., Dixon C. J., Clark A. B., Spaedy O., Skelton A., Edupuganti N., Dzubinski L., Tate H., Dyess G., Lindeman B., Lehmann L. S. (2024). ChatGPT-4 Omni performance in USMLE disciplines and clinical skills: Comparative analysis. JMIR Medical Education.

[B3-ejihpe-16-00023] Buckley T. A., Crowe B., Abdulnour R. E., Rodman A., Manrai A. K. (2025). Comparison of frontier open-source and proprietary large language models for complex diagnoses. JAMA Health Forum.

[B4-ejihpe-16-00023] Chen L., Zaharia M., Zou J. (2023). How is ChatGPT’s behavior changing over time?. arXiv.

[B5-ejihpe-16-00023] Chen Y., Huang X., Yang F., Lin H., Lin H., Zheng Z., Liang Q., Zhang J., Li X. (2024). Performance of ChatGPT and Bard on the medical licensing examinations varies across different cultures: A comparison study. BMC Medical Education.

[B6-ejihpe-16-00023] Cohen J. (1960). A coefficient of agreement for nominal scales. Educational and Psychological Measurement.

[B7-ejihpe-16-00023] Elkin P. L., Mehta G., LeHouillier F., Resnick M., Mullin S., Tomlin C., Resendez S., Liu J., Nebeker J. R., Brown S. H. (2025). Semantic clinical artificial intelligence vs. native large language model performance on the USMLE. JAMA Network Open.

[B8-ejihpe-16-00023] Gilson A., Safranek C. W., Huang T., Socrates V., Chi L., Taylor R. A., Chartash D. (2023). How does ChatGPT perform on the United States Medical Licensing Examination (USMLE)? The implications of large language models for medical education and knowledge assessment. JMIR Medical Education.

[B9-ejihpe-16-00023] Hell B., Trapmann S., Schuler H. (2007). Eine metaanalyse der validität von fachspezifischen studierfähigkeitstests im deutschsprachigen Raum. Empirische Pädagogik.

[B10-ejihpe-16-00023] Ilić D., Gignac G. E. (2024). Evidence of interrelated cognitive-like capabilities in large language models: Indications of artificial general intelligence or achievement?. Intelligence.

[B11-ejihpe-16-00023] ITB-Academic Tests (n.d.). TMS—Test für medizinische Studiengänge.

[B12-ejihpe-16-00023] Kadmon G., Kadmon M. (2016). Academic performance of students with the highest and mediocre school-leaving grades: Does the aptitude test for medical studies (TMS) balance their prognoses?. GMS Journal for Medical Education.

[B13-ejihpe-16-00023] Kung T. H., Cheatham M., Medenilla A., Sillos C., De Leon L., Elepaño C., Madriaga M., Aggabao R., Diaz-Candido G., Maningo J., Tseng V. (2023). Performance of ChatGPT on USMLE: Potential for AI-assisted medical education using large language models. PLoS Digital Health.

[B14-ejihpe-16-00023] Landis J. R., Koch G. G. (1977). The measurement of observer agreement for categorical data. Biometrics.

[B15-ejihpe-16-00023] Li C., Wu W., Zhang H., Li Q., Gao Z., Xia Y., Hernández-Orallo J., Vulić I., Wei F. (2025). 11Plus-Bench: Demystifying multimodal LLM spatial reasoning with cognitive-inspired analysis. arXiv.

[B16-ejihpe-16-00023] Liu M., Okuhara T., Chang X., Shirabe R., Nishiie Y., Okada H., Kiuchi T. (2024). Performance of ChatGPT across different versions in medical licensing examinations worldwide: Systematic review and meta-analysis. Journal of Medical Internet Research.

[B17-ejihpe-16-00023] López Espejel J., Ettifouri E. H., Alassan M. S. Y., Chouham E. M., Dahhane W. (2023). GPT-3.5, GPT-4, or BARD? Evaluating LLMs’ reasoning ability in zero-shot setting and performance boosting through prompts. Natural Language Processing Journal.

[B18-ejihpe-16-00023] Mavrych V., Yaqinuddin A., Bolgova O. (2025). Claude, ChatGPT, Copilot, and Gemini performance versus students in different topics of neuroscience. Advances in Physiology Education.

[B19-ejihpe-16-00023] Meyer A., Riese J., Streichert T. (2024). Comparison of the performance of GPT-3.5 and GPT-4 with that of medical students on the written German medical licensing examination: Observational study. JMIR Medical Education.

[B20-ejihpe-16-00023] Nori H., King N., Mayer McKinney S., Carignan D., Horvitz E. (2023). Capabilities of GPT-4 on medical challenge problems. arXiv.

[B21-ejihpe-16-00023] Schult J., Hofmann A., Stegt S. J. (2019). Leisten fachspezifische studierfähigkeitstests im deutschsprachigen raum eine valide studienerfolgsprognose? Ein metaanalytisches update. Zeitschrift für Entwicklungspsychologie und Pädagogische Psychologie.

[B22-ejihpe-16-00023] Test für Medizinische Studiengänge (TMS) (n.d.). Results and scoring of the test for medical studies (TMS). TMS-info.

[B23-ejihpe-16-00023] TMS (n.d.). Informationen der am TMS beteiligten organisationen.

[B24-ejihpe-16-00023] Trost G. (1992). Erfahrungen mit dem test für medizinische Studiengänge (TMS). Medizinische Ausbildung.

[B25-ejihpe-16-00023] Yang Y., Chen M., Liu Q., Hu M., Chen Q., Zhang G., Hu S., Zhai G., Qiao Y., Wang Y., Shao W., Luo P. (2025). Truly assessing fluid intelligence of large language models through dynamic reasoning evaluation. arXiv.

[B26-ejihpe-16-00023] Zimmerhofer A., Hofmann A., Wittenberg T., Amelung D., Kadmon M. (2019). Test for medical studies (TMS): Testing cognitive competence in applicants for medical school *[Conference presentation]*. 16th DPPD Conference.

[B27-ejihpe-16-00023] Zong H., Wu R., Cha J., Wang J., Wu E., Li J., Zhou Y., Zhang C., Feng W., Shen B. (2024). Large language models in worldwide medical exams: Platform development and comprehensive analysis. Journal of Medical Internet Research.

